# Phytochemical-Mediated Tritrophic Interactions: Effects of Pepper and Eggplant Cultivars on the Green Peach Aphid *Myzus persicae* (Sulzer) and Its Predators

**DOI:** 10.3390/insects16101050

**Published:** 2025-10-15

**Authors:** Zahra Golparvar, Mahdi Hassanpour, Ali Golizadeh, Gadir Nouri Ganbalani, Hooshang Rafiee Dastjerdi, Tomasz Oszako, Mojtaba Hosseini, Stanisław Łuniewski, Mikołaj Jalinik, Ali Chenari Bouket

**Affiliations:** 1 Department of Plant Protection, Faculty of Agriculture and Natural Resources, University of Mohaghegh Ardabili, Ardabil 5619911367, Iran; z.golparvar@uma.ac.ir (Z.G.); golizadeh@uma.ac.ir (A.G.); gnouri@uma.ac.ir (G.N.G.); rafiee@uma.ac.ir (H.R.D.); 2 Forest Research Institute, 3 Braci Leśnej Street, 05-090 Sękocin Stary, Poland; t.oszako@pb.edu.pl; 3 Department of Plant Protection, Faculty of Agriculture, Ferdowsi University of Mashhad, Mashhad 9177948974, Iran; m.hosseini@um.ac.ir; 4 L.N. Gumilyov Eurasian National University, Astana 010008, Kazakhstan; astwa@astwa.pl; 5 Institute of Forest Sciences, Faculty of Civil Engineering and Environmental Sciences, Białystok University of Technology, ul. Wiejska 45E, 15-351 Białystok, Poland; m.jalinik@pb.edu.pl; 6 East Azarbaijan Agricultural and Natural Resources Research and Education Centre, Plant Protection Research Department, Agricultural Research, Education and Extension Organization (AREEO), Tabriz 53551-79854, Iran; a.chenari@areeo.ac.ir

**Keywords:** secondary metabolites, green peach aphid, plant–insect interactions, life history traits, tritrophic interactions

## Abstract

**Simple Summary:**

Plants exhibit various chemical traits that influence their interactions with herbivores and higher trophic levels. In this experiment, we investigated the effects of different host plant cultivars on the life history traits of the green peach aphid and its predators, *Aphidoletes aphidimyza* and *Chrysoperla carnea*. Phytochemical analyses revealed that the eggplant cultivar ‘Longo’ contained the highest concentrations of secondary metabolites and key defensive enzymes. The intrinsic rate of increase (*r*) was lowest for aphids on ‘Longo’, but significantly higher for both predator species when fed aphids from this cultivar. These findings suggest that elevated secondary metabolite levels on ‘Longo’ negatively impact *Myzus persicae* performance while benefiting its predators, highlighting the role of host plant chemistry in shaping tritrophic interactions.

**Abstract:**

The diverse phytochemical profiles of host plants can significantly influence their interactions with herbivores and natural enemies. This study investigated the ‘bottom-up’ effects of several bell pepper and eggplant cultivars on the development, reproduction, and survival of the green peach aphid, *Myzus persicae* (Sulzer) (Hemiptera: Aphididae), and its predators, *Aphidoletes aphidimyza* (Rondani) (Diptera: Cecidomyiidae) and *Chrysoperla carnea* (Stephens) (Neuroptera: Chrysopidae). We analyzed the leaves of each cultivar for levels of total flavonoids, phenols, anthocyanins, and key defensive enzymes. The eggplant cultivar ‘Longo’ exhibited the highest concentration of secondary metabolites. Aphid populations reared on this cultivar’s leaves showed a slower growth rate compared to those on other cultivars. Conversely, predators fed on these aphids demonstrated higher rates of population growth and produced more offspring. Accordingly, the intrinsic rate of natural increase (*r*) was lower for aphids feeding on ‘Longo’, but significantly higher for both *A. aphidimyza* and *C. carnea* when fed those aphids. These results demonstrate that elevated secondary metabolites on ‘Longo’ suppress the performance of *M. persicae* while enhancing predator efficiency, thereby providing a phytochemical-based approach that can serve as an effective component of integrated pest management (IPM) programs.

## 1. Introduction

The green peach aphid, *Myzus persicae* (Sulzer) (Hemiptera: Aphididae), is a highly polyphagous, holocyclic aphid with a worldwide distribution that infests hundreds of plant species [1]. This pest causes significant damage by directly feeding on leaves, flowers, and fruits, thereby reducing host plant yield. It also causes indirect harm by acting as a vector for over 100 plant viruses, such as pepper mottle virus, potato virus Y, and tobacco etch virus [2]. *M. persicae* is an important pest of pepper and eggplant crops in both field and greenhouse settings [3]. While population outbreaks can be managed to some extent with synthetic insecticides [4], the adverse effects of these chemicals on human health and the environment have led to a global shift toward reducing their use [5].

The predacious gall midge, *Aphidoletes aphidimyza* (Rondani) (Diptera: Cecidomyiidae), is an effective biological control agent against many aphid species on field crops, fruit trees [6], greenhouse crops [7] and is commercially utilized as a successful biocontrol agent in several countries [7,8]. Another important predator widely used as an effective biological control agent is the green lacewing, *Chrysoperla carnea* (Stephens) (Neuroptera: Chrysopidae). It preys on a wide range of soft-bodied insects, including aphids, coccids, and insect eggs [9,10,11,12,13]. The multiple biological and ecological traits of this species render it a key component of integrated pest management (IPM) programs [14].

Plant Defensive Mechanisms and Tritrophic Interactions

Plant defenses are activated by mechanical damage, herbivory, environmental stresses [15] synthetic inducers and volatile compounds from neighboring plants [16,17], which can affect herbivore physiology [18]. In response to herbivores, plants produce secondary metabolites, such as phenolics, glucosinolates, nitrogen compounds, and terpenoids [19,20,21], that repel or deter insects [21,22]. Phenols, flavonoids, and anthocyanins are especially important secondary chemicals conferring resistance to phytophagous insects. Environmental stresses can induce the production of reactive oxygen species (ROS) in plants [23,24] that damage cellular macromolecules [25], but plants counteract this with antioxidant systems [26]. Plant antioxidant enzymes such as polyphenol oxidase (PPO), superoxide dismutase (SOD), and catalase (CAT) contribute to their defense by detoxifying ROS [27,28]. PPO generates quinones that bind insect dietary proteins [29,30,31], reducing food quality [31,32], while SOD and CAT act sequentially to neutralize ROS and protect plant cells from oxidative stress [33,34].

Natural enemies play a key role in integrated pest management [35] and can be combined with strategies like host plant resistance. In tritrophic systems, insects must overcome plant defenses, including physical, chemical, and enzymatic barriers [36,37,38]. Studies show that plant compounds and secondary metabolites affect herbivore physiology and antioxidant enzymes, which in turn influence their life history and the performance of predators [36,39,40,41]. Resistance can prolong pest development, increase vulnerability to natural enemies [40,42], and suppress insect immunity such as the phenoloxidase (PO) system [43,44,45], while plant-released volatiles may attract predators indirectly [46]. This highlights the complex plant–herbivore–predator interactions and the importance of plant-mediated biochemical changes for effective and sustainable pest management [37,47,48,49].

Life table parameters, particularly the intrinsic rate of increase (*r*), summarize insect population dynamics and indicate plant resistance [50,51,52]. They also help assess predator prey interactions and prey quality effects on predators [53,54], supporting predictions and planning in pest management. [55,56].

Aim of the Study

This study investigates how different cultivars of eggplant and bell pepper influence the life history of the green peach aphid and its key predators, *A. aphidimyza* and *C. carnea*. Although plant resistance and biological control are well-established IPM tools, the combined effects of host-plant chemistry on both aphids and their natural enemies remain poorly understood. To address this research gap, we quantified secondary metabolites in the tested cultivars and examined their impacts on aphid life table parameters, PO activity, and the survival, development, and reproduction of the predators. These findings provide new insights into plant-mediated tritrophic interactions and their potential to enhance sustainable pest management strategies.

## 2. Materials and Methods

### 2.1. Plants and Insects’ Culture

This study utilized two cultivars of bell pepper (*Capsicum annuum*), ‘California Wonder’ and ‘Hybrid SM’, and two cultivars of eggplant (*Solanum melongena*), ‘Longo’ and ‘Kemer’. These cultivars were selected due to their widespread production in Iranian greenhouses. Seeds were planted in 20 cm diameter plastic pots containing a sterilized mixture of soil, sand, and cattle manure (2:2:1). Plants were grown in an aphid-free greenhouse under controlled conditions of 25 ± 3 °C, 60 ± 5% relative humidity (RH), and a 14:10 (L:D) h photoperiod. All experiments were conducted when plants reached the six-leaf stage. For secondary metabolite measurement, the second fully expanded leaf from the top of each plant was collected.

A laboratory colony of *M. persicae* was obtained from the plant protection laboratory at the University of Tabriz, Iran. Before the experiments, aphids were reared for two generations on each of the four plant cultivars.

Larvae of *A. aphidimyza* were collected from aphid-infested pepper plants in a greenhouse in Ardabil, Iran. Prior to life table experiments, the predator larvae were reared for three generations in transparent plastic cages (45 × 30 × 45 cm) covered with a fine mesh for aeration, with each cage containing *M. persicae*-infested plants.

The *C. carnea* colony was established from a laboratory culture at the plant protection laboratory, University of Tabriz, Iran. Adults were reared in similar plastic cages and fed an artificial diet composed of brewer’s yeast, honey, and distilled water in a 4:7:5 ratio [57]. Eggs were collected from cages and transferred with a fine brush to ventilated plastic containers (17.5 cm diameter; 7.5 cm height) for hatching. Predator larvae were reared for three generations before the experiments.

All insect colonies were maintained in a growth chamber at 25 ± 1 °C, 60 ± 5% RH, and a 16:8 (L:D) h photoperiod.

### 2.2. Life Table Parameters

To estimate life table parameters of *M. persicae*, around 50 apterous adults were individually confined within leaf clip-cages (2 cm diameter × 1 cm height) on host plant leaves. To obtain same-aged nymphs, all adults were removed after 24 h, leaving a single first-instar nymph per cage. Experiments were conducted in a growth chamber at 25 ± 1 °C, 65 ± 5% RH, and a 16:8 (L:D) h photoperiod. Each aphid was monitored daily with a 10× magnifying glass to record molting (exuviae as an indicator of molting to the next instar) and survival. Upon reproduction onset, offspring numbers and adult mortality were recorded daily; offspring were removed every 24 h until all adults died.

Newly hatched larvae of *A. aphidimyza* were individually transferred into transparent plastic containers (8 × 7 × 4 cm) with ventilated tops covered by a fine-mesh net. Each container contained infested leaves from one of the four host plant cultivars (*n* = 60 per treatment). Larvae were examined daily for developmental stage and mortality. Since *A*. *aphidimyza* larvae drop to the ground for pupation [58], a 2 cm layer of autoclaved (120 °C, 20 min) moist fine sand was added to containers. Upon adult emergence, 20 pairs of midges were transferred to separate containers (11 cm diameter × 9.5 cm height) with mesh tops for aeration. Approximately 50 aphid nymphs were provided per experimental unit to ensure reproduction. Adult fecundity and mortality were recorded daily, with eggs counted and removed every 24 h until adult death.

To assess *C. carnea* performance, 60 same-aged predator eggs from the stock culture were prepared per host plant cultivar. Eggs were placed individually in plastic Petri dishes (8 × 7 × 4 cm) with ventilated lids and moistened filter paper bottoms. Upon hatching, larvae were fed infested leaves of each cultivar daily with sufficient *M. persicae* supply. Larval development and mortality were recorded daily. After maturity, 20 pairs of adult predators were mated and housed individually in transparent cages. Eggs laid by females were collected and counted daily. Mean female progeny per adult lifetime were recorded as life table parameters.

### 2.3. Bell Pepper and Eggplant Metabolite Assay

#### 2.3.1. Total Phenolic Compounds

Phenolic compounds in leaf extracts were quantified by the Folin–Ciocalteu method [59]. Methanolic leaf extract (0.1 mL) was added to 2.5 mL of 10-fold diluted Folin–Ciocalteu reagent, followed by 2 mL of 7.5% sodium carbonate. Samples were incubated at 40 °C for 30 min. Absorbance was measured by UV–Vis spectrophotometer at 760 nm. Results were expressed as gallic acid equivalents (mg GAE/g FW).

#### 2.3.2. Flavonoids

Flavonoid content was determined following Mashabela et al. (2015) [60]. One gram of leaves was homogenized in 10 mL ethanol, then 225 µL of 5% sodium nitrite was added to 375 µL of extract. After 5 min, 450 µL of 10% aluminum chloride was added, followed by 1.5 mL of 1 M sodium hydroxide after 6 min. Absorbance was read at 510 nm by using a UV–Vis spectrophotometer (SP-UV 300, Spectrum Instruments, Shanghai, China) and expressed as quercetin equivalents (mg QE/g FW).

#### 2.3.3. Anthocyanin

Dried leaf powder (1 g) was extracted with 10 mL 70% methanol containing 0.2% formic acid, shaken for 40 min, centrifuged at 5000 rpm for 20 min at 4 °C; supernatant was collected and extraction repeated with 5 mL solvent [61]. Anthocyanin content was measured by pH differential method, absorbance recorded at 510 nm by using a UV–Vis spectrophotometer [62], expressed as delphinidin-3-glucoside equivalents (mg DGE/g DP).

#### 2.3.4. Polyphenol Oxidase (PPO)

PPO activity was measured according to Zhang et al. (2008) [63]. Leaves (0.5 g) were homogenized in phosphate buffer (0.05 M, pH 5.8) containing 5% PVP. Homogenate was filtered and centrifuged (10,000× *g*, 15 min, 4 °C), and the supernatant was re-centrifuged for enzymatic extract. Reaction mixture: 3.9 mL phosphate buffer (0.05 M, pH 5.3), 0.1 M catechol, 0.5 mL enzymatic extract. The absorbance change at 525 nm was recorded for 3 min at 20 s intervals by a UV–Vis spectrophotometer; results were expressed as ∆525 min^−1^ g^−1^ protein.

#### 2.3.5. Superoxide Dismutase (SOD)

SOD activity was assayed in leaves (1 g) homogenized in sodium phosphate buffer (0.05 M, pH 7.8) with 1 mM EDTA and 2% PVP. The supernatant was centrifuged at 13,000× *g* for 20 min (4 °C). Reaction mix: methionine (13 mM), riboflavin (1.3 µM), sodium carbonate (0.05 M, pH 10.2), NBT (63 µM) and 0.5 mL extract. Mixtures were illuminated for 15 min; absorbance was read by UV–Vis spectrophotometer at 560 nm. Results corresponded to an enzyme amount causing 50% inhibition of NBT reduction [64].

#### 2.3.6. Catalase (CAT)

Leaves (2.5 g) were homogenized in 25 mM sodium phosphate buffer (pH 7.8) with 1 mM EDTA and PVP and centrifuged (12,000× *g*, 20 min, 4 °C). The supernatant was used for assay with phosphate buffer (pH 7.0), 40 mM H_2_O_2_, and enzymatic extract. Decomposition of H_2_O_2_ was measured at 240 nm for 3 min at 20 s intervals by a UV–Vis spectrophotometer [65].

### 2.4. Determination of Phenoloxidase (PO) Activity in Aphid Hemolymph

#### 2.4.1. Hemolymph Collection

Adult aphids were transferred to 1 mL distilled water, homogenized, and centrifuged at 13,000× *g* for 15 min at 4 °C; the procedure was repeated 4 times.

#### 2.4.2. PO Preparation

Approximately 40 µL hemolymph was mixed with anticoagulant solution (0.01 M EDTA, 0.1 M glucose, 0.062 M NaCl, 0.026 M citric acid, pH 4.6) in 4:5 ratio. The mixture was centrifuged (12,000× *g*, 5 min) [66] and the pellets washed twice in phosphate buffer (pH 6.5) [67]. A total of 500 µL cold phosphate buffer was added to pellet, homogenized, and centrifuged (12,000× *g*, 15 min). Hemocyte lysate supernatant was collected. Samples were incubated for 30 min at 30 °C; 50 µL L-DOPA was added and the samples were incubated for 30 min more. Absorbance change was measured by using UV–Vis spectrophotometer at 490 nm/min every 30 s for 2 min. One enzyme unit was defined as an increase in absorbance of 0.01 min^−1^ [68].

#### 2.4.3. Protein Content Determination

Total PO protein was measured by bovine serum albumin (Bio-Rad) standard [69].

### 2.5. Statistical Analysis

Life history data of *M. persicea* and its predators were analyzed using the age-stage, two-sex life table approach implemented in TWOSEX-MSChart program (version 2018.11.15) [70,71]. To determine the mean values and standard errors of life table parameters, a bootstrap method with 100,000 iterations was applied [72]. Pairwise comparisons among the four treatments were conducted using a paired bootstrap test with a significance level of *p* < 0.05 (Chi, 2015) [71]. Based on this method, the age-stage-specific survival rate (*s_xj_*), where *x* represents age and *j* represents stage, along with age-specific survival (*l_x_*) and fecundity (*m_x_*), were calculated as follows:

(1) $$s_{xj}=\frac{n_{xj}}{n_{01}}$$

The term *n*_01_ refers to the total count of individuals in the initial cohort, and *n_xj_* indicates the number of individuals that survived to a given age *x* and stage *j*.

(2) $$l_{x}=\sum_{j=1}^{k}s_{xj}$$

and

(3) $$m_{x}=\frac{\sum_{j=1}^{k}s_{xj}f_{xj}}{\sum_{j=1}^{k}s_{xj}}$$

where *K* represents the number of developmental stages. Among life-history parameters, the intrinsic rate of increase is considered the most important and was calculated using the Euler–Lotka equation, beginning from age 0 [73].

(4) $$\sum_{x=0}^{\omega }l_{x}m_{x}e^{-r\left(x+1\right)}=1$$

In addition, other life table parameters, including net reproductive rate (*R*_0_), finite rate of increase (*λ*), mean generation time (*T*), were calculated.

Nested ANOVA was used to assess the effects of host plant species (bell pepper vs. eggplant) and cultivars nested within species on life table parameters of *M. persicae* and predators. Secondary metabolites and enzyme activities of host plants were also analyzed using nested ANOVA. Estimations were conducted using R project for statistical computing version 4.2.0 (R Core Team, 2022) [74].

Antioxidant compounds were analyzed in a completely randomized design (CRD) with two groups (aphid-infested and non-infested) and three replications. One-way ANOVA followed by Tukey’s test (5% significance) was used. For each host plant cultivar, independent *t*-tests were conducted to evaluate changes in secondary metabolite and defensive enzyme activities before and after infestation. Prior to the *t*-tests, data normality were verified using the Shapiro–Wilk test. All statistical analyses were conducted using IBM SPSS statistics 26 software. Mean comparisons were performed using Tukey’s Honestly Significant Difference (HSD) test at *p* = 0.05. Figures were generated in SigmaPlot 12.5.

## 3. Results

### 3.1. Development, Survival, and Fecundity of M. persicae

The total preadult (*F*_2,179_ = 12.54, *p* < 0.0001), adult longevity (*F*_2,177_ = 1.9, *p* < 0.001) and fecundity (*F*_2,177_ = 20.23, *p* < 0.0001) of *M. persicae* were significantly affected by host plant cultivars but not by species (bell pepper vs. eggplant). A similar pattern was observed for the life table parameters of *M. persicae* such as the net reproductive rate (*R*_0_) (*F*_2,177_ = 43.88, *p* < 0.0001),the intrinsic rate of increase (*r*) (*F*_2,177_ = 85.12, *p* < 0.0001), the finite rate of increase (*λ*) (*F*_2,177_ = 84.06, *p* < 0.0001) and the mean generation time (*T*) (*F*_2,177_ = 46.84, *p* < 0.0001).

The developmental time, longevity, and fecundity of *M. persicae* adults reared on the different cultivars are presented in Table 1. The nymphal developmental time of *M. persicae* on the eggplant cultivar ‘Longo’ was significantly longer than on all other cultivars. Conversely, the shortest developmental period was observed on the eggplant cultivar ‘Kemer’. Aphids reared on ‘Longo’ also exhibited the shortest adult longevity and the lowest fecundity.

**Figure 1:**
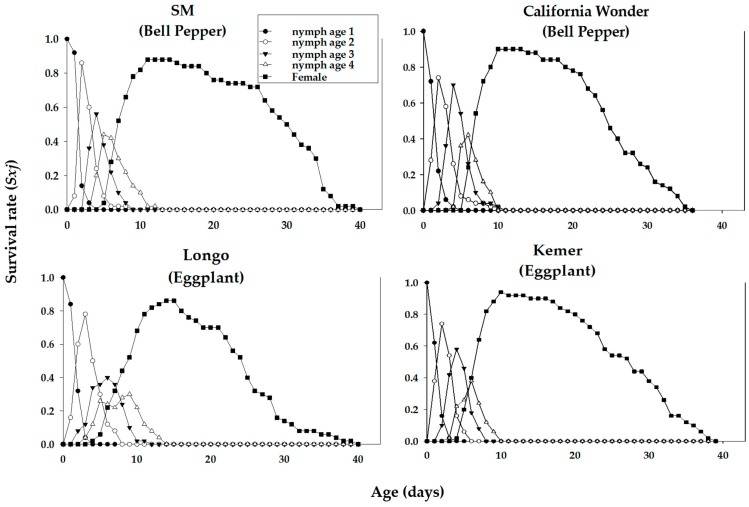
Age-stage-specific survival rate (*s_xj_*) of the green peach aphid, *Myzys persicae*, reared on four different cultivars of bell pepper and eggplant plants.

**Figure 2:**
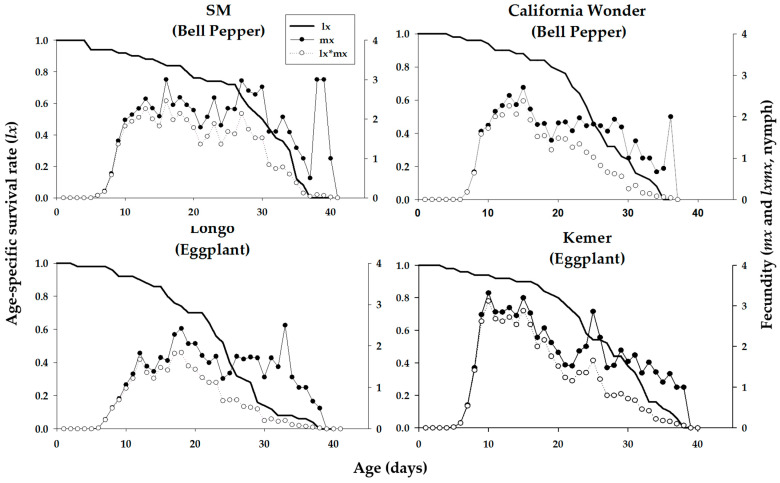
Age-specific survival rate (*l_x_*), age-specific fecundity (*m_x_*), and age-specific maternity (*l_x_m_x_*) of *Mysus persicae* reared on four different cultivars of bell pepper and eggplant plants.

Age-stage-specific survival rates (*s_xj_*) of *M. persicae* on various cultivars are plotted in Figure 1. These curves indicate the probability that a newborn aphid survives to age *x* and stage *j* on different cultivars examined. Significant stage overlapping was observed in s_xj_ curves due to variation in the development rate that occurred among individuals. ‘Longo’ showed the lowest age-stage specific survival rate of nymph and adult stages of *M. persicae.* The age-specific survival rate (*l_x_*), fecundity (*m_x_*), and net maternity (*l_x_m_x_*) curves of *M. persicae* on different cultivars of bell pepper and eggplant are shown in Figure 2. The *l_x_* curve indicates that the death of the last female of *M. persicae* occurred on days 36, 34, 37, and 37 on the bell pepper cultivars ‘SM’ and ‘California Wonder’ and eggplant cultivars ‘Longo’ and ‘Kemer’, respectively. *Myzus persicae* adults produced maximum offspring, namely 16, 15, 32, and 9 at the ages of 3, 2.7, 2.5, and 3.3 on the mentioned cultivars, respectively (Figure 2).

The life table parameters of *M. persicae* were significantly affected by different host plant cultivars (Table 2). The highest and lowest net reproductive rates (*R*_0_) were observed on the bell pepper cultivar ‘SM’ and eggplant cultivar ‘Longo’, respectively. The highest intrinsic rate of increase (*r*), finite rate of increase (*λ*), and shortest mean generation time (*T*) were obtained on the eggplant cultivar ‘Kemer’. In contrast, the lowest values of *r* and *λ* were recorded on ‘Longo’, with the longest generation time observed on ‘SM’.

### 3.2. Development, Survival, and Fecundity of A. aphidimyza

The effects of different host plant species and cultivars on the development and life table parameters of *A. aphidimyza* were estimated by nested ANOVA. The results revealed significant differences among cultivars for the total preadult (*F*_2,162_ = 8.34, *p* < 0.0001), female longevity (*F*_2,75_ = 1.62, *p* < 0.05), male longevity (*F*_2,43_ = 2.05, *p* < 0.05), fecundity (*F*_2,75_ = 3.59, *p* < 0.05), and life table parameters among which *R*_0_ (*F*_2,75_ = 23.42, *p* < 0.0001, *r* (*F*_2,75_ = 29.86 *p* < 0.0001), *λ* (*F*_2,75_ = 30.13, *p* < 0.0001), and *T* (*F*_2,75_ = 11.62, *p* < 0.0001).

The longest immature developmental period was recorded on the eggplant cultivar ‘Kemer’ and the shortest on eggplant cultivar ‘Longo’ (Table 3). Both male and female *A. aphidimyza* lived longer as adults when feeding on aphids reared on ‘Longo’ (Table 3).

These results indicate that the host plant cultivar influences the developmental timing and longevity of *A. aphidimyza* through effects mediated via the aphid prey.

*Aphidoletes aphidimyza* fed on aphids maintained on different host plant cultivars exhibited significant differences in the adult pre-oviposition period (APOP), total pre-oviposition period from emergence to first oviposition (TPOP), oviposition period, and fecundity (Table 4). The oviposition period ranged from 4.21 to 5.31 days, with the longest duration observed in individuals fed on aphids cultured on the eggplant cultivar ‘Longo’ and the shortest on cultivar ‘Kemer’. Likewise, fecundity was highest on ‘Longo’ and lowest on ‘Kemer’.

**Figure 3:**
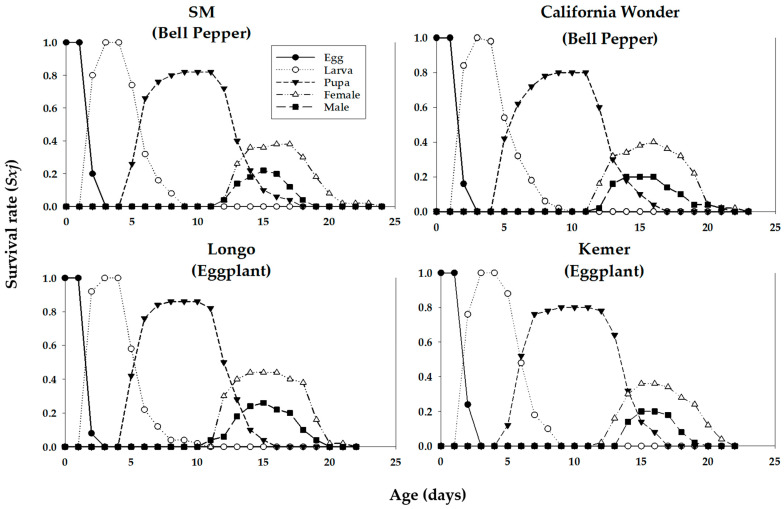
Age-stage-specific survival rate (*s_xj_*) of *Aphidoletes aphidimyza* fed on *Myzus persicae* reared on four different cultivars of bell pepper and eggplant plants.

**Figure 4:**
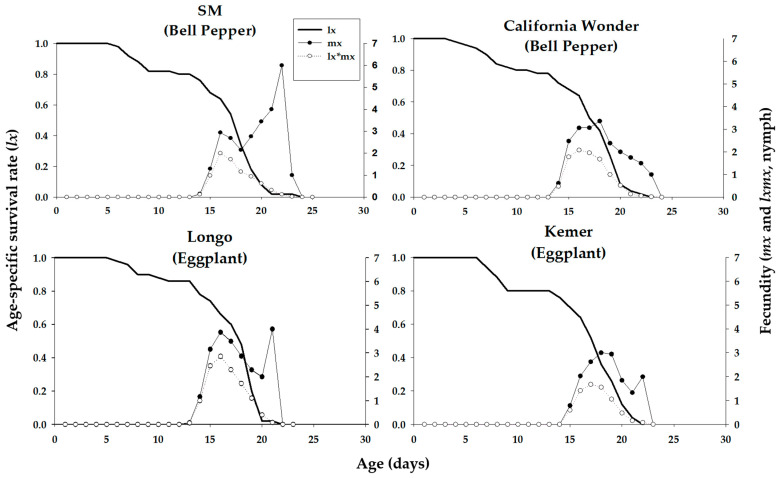
Age-specific survival rate (*l_x_*), age-specific fecundity (*m_x_*), and age-specific maternity (*l_x_m_x_*) of *Aphidoletes aphidimyza* fed on *Myzus persicae* fed on four different cultivars of bell pepper and eggplant plants.

The age-stage-specific survival rate (*s_xj_*) of *A. aphidimyza* on different host plant cultivars was observed to overlap among stages due to the variation in developmental rates among individuals (Figure 3). The highest survival rate of *A. aphidimyza* adults was on the eggplant cultivar Longo. Fecundity (*m_x_*), *l_x_*, and *l_x_m_x_* of *A. aphidimyza* fed on *M. persicae* reared on different cultivars of bell pepper and eggplant are shown in Figure 4.

The population parameters of *A. aphidimyza* were significantly influenced by the host plant cultivar on which the aphids were reared (Table 5).

The highest values of *R*_0_, *r*, and *λ* were all obtained when the predator consumed aphids from the eggplant cultivar ‘Longo’. Conversely, the lowest values for these parameters were observed on aphids from the cultivar ‘Kemer’. Furthermore, *A. aphidimyza* reared on aphids from ‘Longo’ exhibited the shortest *T* compared to all other cultivars.

These results highlight that host plant cultivar indirectly affects predator population growth by influencing the quality of their aphid prey.

### 3.3. Development, Survival, and Fecundity of C. carnea

The total preadult (*F*_2,153_ = 23.08, *p* < 0.0001), female longevity (*F*_2,70_ = 6.7, *p* < 0.01), male longevity (*F*_2,65_ = 4.76, *p* < 0.05), fecundity (*F*_2,75_ = 3.59, *p* < 0.05), and life table parameters of *C. carnea* including *R*_0_ (*F*_2,70_ = 50.926, *p* < 0.0001), r (*F*_2,70_ = 14.40 *p* < 0.0001), *λ* (*F*_2,70_ = 33.07, *p* < 0.0001), and *T* (*F*_2,70_ = 13.86, *p* < 0.0001) were significantly affected by the host plant cultivar on which the aphid prey were reared.

The immature developmental period and adult longevity of *C. carnea* also differed significantly among predators reared on aphids fed on various host cultivars (Table 6). The shortest immature developmental period was recorded on bell pepper cultivar ‘California Wonder’ (20.05 ± 0.2 days). Female *C. carnea* lived longer when fed aphids from the eggplant cultivar ‘Longo’ (65.99 ± 1.6 days), and male longevity was also prolonged when fed aphids from ‘Longo’ (59.71 ± 1.9 days) and ‘California Wonder’ (59.77 ± 1.9 days).

These results indicate that the quality of aphid prey, influenced by the host plant cultivar, significantly affects the biological performance and population parameters of *C. carnea*.

The results of the effects of different host plant cultivars on APOP, TPOP, oviposition period, and fecundity of *C. carnea* are shown in Table 7. The TPOP of *C. carnea* ranged from 23.35 to 26.54 days and was longest when fed on aphid reared on the bell pepper cultivar ‘SM’ and lowest when fed on aphids reared on the bell pepper cultivar ‘California Wonder’. Also, APOP was the lowest on ‘SM’ (3.55 ± 0.1 days) and eggplant cultivar ‘Longo’ (3.56 ± 0.1 days). When fed on aphids reared on ‘Longo’, *C. carnea* females had the longest oviposition period (38.10 ± 1.6 days) and the highest fecundity (958.12 ± 58.8 offspring per female).

**Figure 5:**
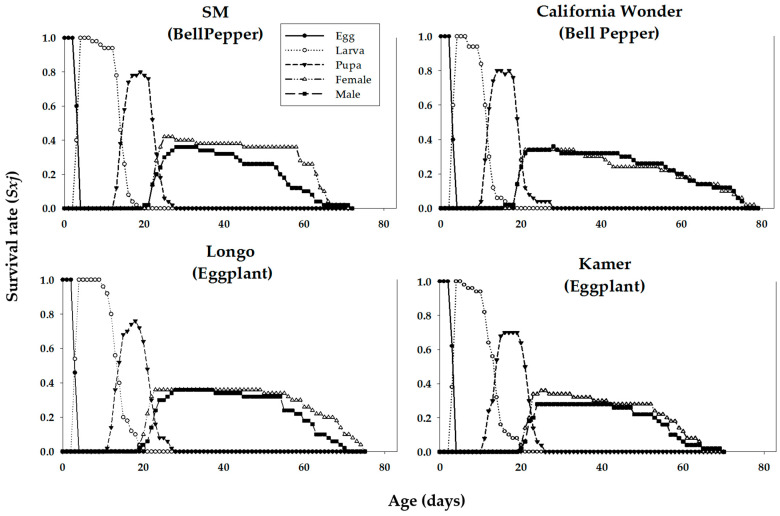
Age-stage-specific survival rate (*s_xj_*) of *Chrysoperla carnea* fed on *Myzus persicae* reared on four different cultivars of bell pepper and eggplant plants.

**Figure 6:**
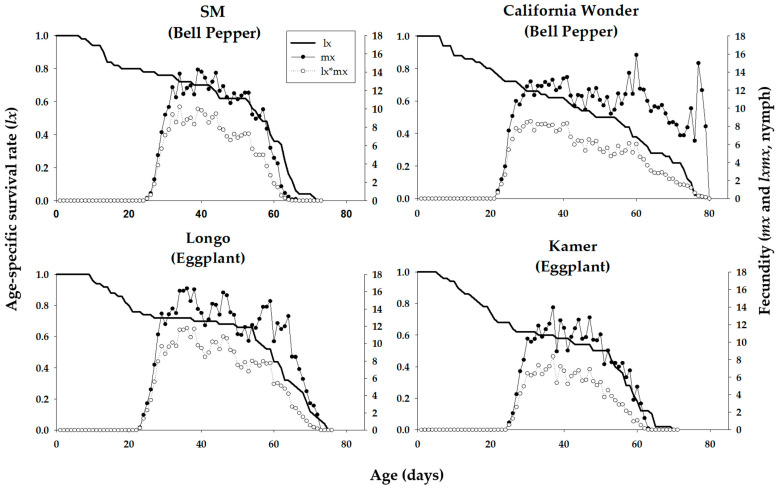
Age-specific survival rate (*l_x_*), age-specific fecundity (*m_x_*), and age-specific maternity (*l_x_m_x_*) of *Chrysoperla carnea* fed on *Myzus persicae* fed on four different cultivars of bell pepper and eggplant plants.

The age-stage-specific survival rate (*s_xj_*), *m_x_*, *l_x_*, and *l_x_m_x_* of *C. carnea* consuming *M. persicae* reared on different cultivars of bell pepper and eggplant are shown in Figure 5 and Figure 6, respectively.

The life table parameters of *C. carnea* were significantly influenced by the host plants on which the aphids were reared (Table 8).

The highest *r* and *λ* were recorded when *C. carnea* fed on aphids from the bell pepper cultivar ‘California Wonder’ (0.159 ± 0.007 day^−1^ and 1.173 ± 0.008 day^−1^, respectively) and eggplant cultivar ‘Longo’ (0.157 ± 0.006 day^−1^ and 1.170 ± 0.007 day^−1^, respectively). Conversely, the lowest values for these parameters occurred on the eggplant cultivar ‘Kemer’ (0.139 ± 0.006 day^−1^ and 1.149 ± 0.007 day^−1^).

The shortest *T* was observed on ‘California Wonder’ (35.21 ± 0.7 days), while the highest *R*_0_ was noted on ‘Longo’ (344.49 ± 68.12 offspring) (Table 8).

These results suggest that the quality of aphid prey, influenced by host plant cultivar, impacts population growth parameters of *C. carnea*, thereby affecting its potential as a biological control agent.

### 3.4. Biochemical Compounds of Host Plants

The concentrations of secondary compounds in the four host plant cultivars were compared under aphid-infested and non-infested conditions (Table 9). Following infestation by *M. persicae*, total phenolic contents increased significantly in bell pepper cultivar ‘SM’ (*t* = −4.882, *df* = 4, *p* < 0.01) and eggplant cultivars ‘Longo’ (*t* = −6.648, *df* = 4, *p* < 0.01) and ‘Kemer’ (*t* = −6.116, *df* = 4, *p* < 0.01). Flavonoid (bell pepper cultivar ‘California Wonder’: ( *t*= −5.215, *df* = 4, *p* < 0.01); ‘Longo’: (*t* = −3212, *df* = 4, *p* < 0.05); ‘Kemer:’ (*t* = −3.637, *df* = 4, *p* < 0.01)) and anthocyanin (‘SM’: (*t* = −6.261, *df* = 4, *p* < 0.01); ‘California Wonder’: (*t* = −6.001, *df* = 4, *p* < 0.01); ‘Longo’: (*t* = −4.780, *df* = 4, *p* < 0.01); ‘Kemer’: (*t* = −3.637, *df* = 4, *p* < 0.05)) contents also showed significant increases across all cultivars after aphid infestation.

Additionally, separate assays of antioxidant compounds revealed that the activities of PPO on ‘SM’ (*t* = −3.674, *df* = 4, *p* < 0.05), ‘California Wonder’ (*t* = −7.209, *df* = 4, *p* < 0.01), and ‘Longo’ (*t* = −8.280, *df* = 4, *p* < 0.05) and ‘Kemer’ (*t* = −9.096, *df* = 4, *p* < 0.05), SOD on ‘SM’ (*t* = −19.989, *df* = 4, *p* < 0.01), ‘California Wonder’ (*t* = −14.576, *df* = 4, *p* < 0.01) and ‘Longo’ (*t* = −15.288, *df* = 4, *p* < 0.01) and ‘Kemer’ (*t* = −8.482, *df* = 4, *p* < 0.01), and CAT in ‘SM’ (*t* = −4.817, *df* = 4, *p* < 0.05), ‘California Wonder’ (*t* = −18.441, *df* = 4, *p* < 0.01), ‘Longo’ (*t* = −15.092, *df* = 4, *p* < 0.01), and ‘Kemer’ (*t* = −4.060, *df* = 4, *p* < 0.05) were significantly higher in all infested plants compared to their non-infested counterparts (Table 10).

Nested ANOVA revealed no significant differences between host plant species (bell pepper vs. eggplant) in relation to chemical compound concentrations, except for PPO activity, which was significantly higher in cultivar Longo before (*F*_1,8_ = 24.5, *p* < 0.05) and after (*F*_1,8_ = 31.82, *p* < 0.05) infestation. However, significant differences were observed among host plant cultivars for both secondary metabolites (phenolic contents: (*F*_2,8_ = 48.74, *p* < 0.001); flavonoid: (*F*_2,8_ = 155.5, *p* < 0.001), anthocyanin: (*F*_2,8_ = 421.7, *p* < 0.001)) and defensive enzymes (PPO: (*F*_2,8_ = 0.66, *p* < 0.05); SOD: (*F*_2,8_ = 67.16, *p* < 0.001), CAT: (*F*_2,8_ = 832.18, *p* < 0.001)). Subsequently, following aphid infestation, significant variation in secondary metabolites (phenolic contents: (*F*_2,8_ = 45.55, *p* < 0.001); flavonoid: (*F*_2,8_ = 42.36, *p* < 0.001), anthocyanin: (*F*_2,8_ = 76.01, *p* < 0.001)) and defensive enzymes (PPO: (*F*_2,8_ = 8.01, *p* < 0.05); SOD: (*F*_2,8_ = 478.18, *p* < 0.001), CAT: (*F*_2,8_ = 110.00, *p* < 0.001)) were detected among host plant cultivars.

The concentrations of secondary metabolites in aphid-infested leaves differed significantly among the studied cultivars (Table 9). Total phenolic content varied significantly among cultivars (*F*_4,11_ = 32.53; *p* < 0.01), with the lowest level found in the eggplant cultivar ‘Kemer’ and the highest in the bell pepper cultivar ‘Colifornia Wonder’.

Flavonoid concentrations in leaf extracts ranged from 68.93 to 170.47 mg/g (*F*_4,11_ = 8.44; *p* < 0.01), with the highest content observed in ‘Longo’ and the lowest in ‘Kemer’. Anthocyanin content also differed significantly among cultivars (*F*_4,11_ = 21.34; *p* < 0.01), with the highest and lowest values recorded in the cultivars ‘Longo’ and ‘Kemer’, respectively.

### 3.5. Activities of Defensive Enzymes in Non-Infested Leaves

The activities of defensive enzymes in non-infested bell pepper and eggplant leaves are presented in Table 10. Polyphenol oxidase (PPO) activity (*F*_4,11_ = 5.89; *p* < 0.05) ranged from 0.00263 to 0.00602 µmol/mg protein/min, with the lowest activity recorded in the bell pepper cultivar ‘California Wonder’ and the highest in eggplant cultivar ‘Longo’. Superoxide dismutase (SOD) levels were also significantly higher in non-infested leaves of cultivar ‘Longo’ (*F*_4,11_ = 50.39; *p* < 0.01). Catalase (CAT) activity showed a significant effect of cultivar (*F*_4,11_ = 70.88; *p* < 0.05), with the highest activity found in ‘Longo’ and the lowest in the cultivar ‘Kemer’.

### 3.6. Effects of Different Host Plants on PO Activity of M. persicae

**Figure 7:**
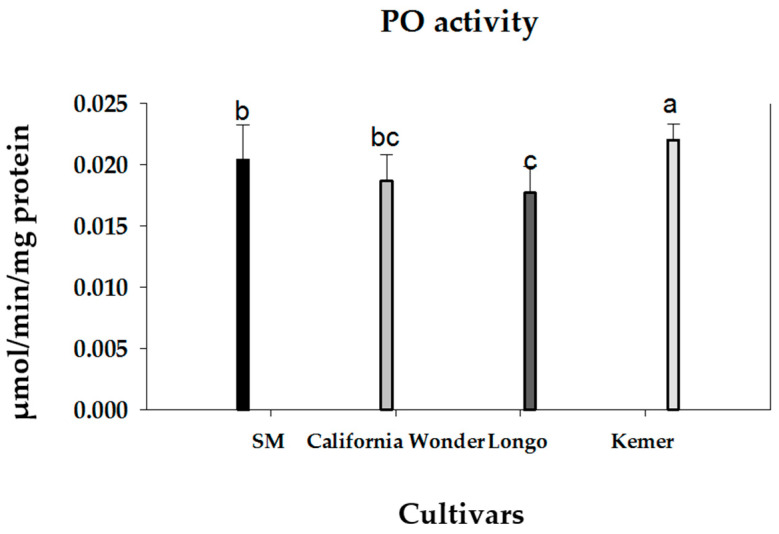
The effect of four bell pepper and eggplant cultivars on the activity of hemocyte-derived phenoloxidase in the green peach aphid, *Myzus persicae*. Data are shown as the mean ± standard errors. Different letters above the bars indicate significant differences among treatments (Tukey’s test, *p* < 0.05).

The effects of host plant nutrition on PO activity in *M. persicae* are illustrated in Figure 7. Phenoloxidase activity was significantly influenced by the host plant cultivar (*F*_4, 11_ = 2.36; *p* < 0.05), with the lowest enzyme activity observed in aphids reared on eggplant cultivar ‘Longo’ and the highest in those from cultivar ‘Kemer’.

## 4. Discussion

Different host plants and cultivars provide varying levels of resistance to pest species. Qualitative traits, such as specific allelochemicals and phenological changes, influence insect development, survival, and reproduction and also affect natural enemies [50,75,76,77]. Therefore, understanding the population dynamics of both pests and their natural enemies across different host plants is essential for effective pest management [78,79].

Our analysis showed that host plant cultivar significantly affected the performance of *M. persicae* and its predators (*A. aphidimyza* and *C. carnea*). Differences among cultivars had a greater impact on aphid life table parameters than differences between species. Aphids reared on the eggplant cultivar ‘Longo’ exhibited prolonged developmental time, increasing their exposure to predators and thus their susceptibility to attack. These variations likely arise from differences in secondary metabolite content, which inhibit aphid growth and reproduction.

The intrinsic rate of increase (r) reflects developmental time, sex ratio, time to first reproduction, and daily fecundity [50,80]. *Myzus persicae* reared on the cultivar ‘Longo’ had the lowest r, consistent with elevated levels of phenols, flavonoids, and anthocyanins in this cultivar, which confer antibiosis resistance. Aphid feeding further stimulated production of these compounds, consistent with previous reports [49,81,82,83].

Secondary metabolites in ‘Longo’ are associated with enhanced PPO activity and increased antioxidant enzyme activities (CAT and SOD), which help manage ROS generated during herbivory. This combination of chemical defenses likely contributes to reduced aphid growth and lower PO activity, indirectly enhancing predator performance. In contrast, the eggplant cultivar ‘Kemer’ showed high PPO but low SOD and CAT activities, indicating less effective ROS detoxification and higher aphid susceptibility. Quinones produced via PPO-mediated oxidation of phenols act directly as insect toxins [84].

Delays in herbivore development caused by plant defenses increased pest vulnerability to natural enemies, reflected in predator life table parameters. Flavonoid residues ingested via prey may further stimulate predator reproduction [49,85]. Eggplant cultivar ‘Longo’, with high chemical defenses, affected the survival, longevity, and fecundity of both *A. aphidimyza* and *C. carnea*. The highest r and λ for *A. aphidimyza* were recorded when feeding on aphids from ‘Longo’. Similarly, *C. carnea* performed best on ‘Longo’ and the bell pepper cultivar ‘California Wonder’, with no significant difference between them.

Overall, host plant resistance modulates both pest and predator performance through a combination of direct effects on aphid physiology and indirect effects mediated via compromised prey immunity. Aphids from cv. ‘Longo’ with elevated biochemical defenses exhibited lower PO activity, potentially contributing to enhanced predator efficiency [43,44,86].

## 5. Conclusions

Our study of tritrophic interactions among different host plant cultivars, *M. persicae*, *A. aphidimyza*, and *C. carnea* revealed that the performance of green peach aphids is directly influenced by the host plant. This effect, in turn, significantly impacts the growth and development of their predators. We found that host plants with high levels of secondary metabolites, such as the eggplant cultivar ‘Longo’, exhibit enhanced resistance to herbivore attacks while also improving the efficacy of biological control agents.

Based on these results, populations of *M. persicae* in greenhouses can be effectively managed within an Integrated Pest Management framework. This approach combines the cultivation of resistant cultivars, like the cultivar ‘Longo’, with the strategic release of natural enemies, such as *A. aphidimyza* and *C. carnea*. Implementing this strategy can reduce pest populations, enhance predator performance, and contribute to sustainable and environmentally friendly pest management.

## Figures and Tables

**Table 1:** Mean (±SE) developmental times (days), adult longevity, and fecundity of the green peach aphid, *Myzus persicae*, reared on four different cultivars of bell pepper and eggplant.

	Nymph Developmental Time (day)						
Cultivar	1st Instar	2nd Instar	3rd Instar	4th Instar	Total Preadult	Adult Longevity (day)	Fecundity (Nymph/Female)
SM (Bell Pepper)	2.10 ± 0.0 a	1.82 ± 0.0 b	1.80 ± 0.0 b	1.91 ± 0.1 a	7.63 ± 0.1 b	22.20 ± 0.9 a	49.37 ± 3.8 a
California Wonder (Bell Pepper)	2.02 ± 0.1 ab	2.06 ± 0.1 b	2.13 ± 0.1 a	1.46 ± 0.0 b	7.67 ± 0.1 b	18.80 ± 0.8 b	36.09 ± 2.4 b
Longo (Eggplant)	2.18 ± 0.1 a	2.52 ± 0.1 a	2.06 ± 0.1 ab	2.02 ± 0.1 a	8.78 ± 0.3 a	16.70 ± 1.0 b	29.13 ± 2.8 b
Kemer (Eggplant)	1.75 ± 0.0 b	1.87 ± 0.1 b	1.85 ± 0.1 ab	1.38 ± 0.0 b	6.85 ± 0.2 c	20.76 ± 1.0 a	47.52 ± 3.6 a

Means within each column followed by different letters are significantly different (paired bootstrap test; *p* < 0.05).

**Table 2:** Mean (±SE) life table parameters of the green peach aphid, *Myzus persicae*, reared on four different cultivars of bell pepper and eggplant.

Parameter (Mean ± SE)				
Cultivar	*R*_0_ (Offspring)	*r* (day ^−1^)	*λ* (day ^−1^)	*T* (day)
SM (Bell Pepper)	44.43 ± 4.03 a	0.249 ± 0.008 b	1.282 ± 0.001 b	15.23 ± 0.3 a
California Wonder	32.48 ± 2.66 b	0.248 ± 0.008 b	1.281 ± 0.001 b	14.02 ± 0.3 b
(Bell Pepper)				
Longo (Eggplant)	25.64 ± 2.81 b	0.216 ± 0.009 c	1.241 ± 0.001 c	14.95 ± 0.3 ab
Kemer (Eggplant)	44.67 ± 3.75 a	0.290 ± 0.009 a	1.337 ± 0.001 a	13.07 ± 0.2 c

Means within each column followed by different letters are significantly different (paired bootstrap test; *p* < 0.05). *R*_0_: net reproductive rates; *r*: intrinsic rate of increase; *λ*: finite rate of increase; *T*: generation time.

**Table 3:** Mean (±SE) developmental times (days) of immature and adult stages of *Aphidoletes aphidimyza* fed on *Myzus persicae* reared on four different cultivars of bell pepper and eggplant.

	Immature Developmental Time (day)				Adult Longevity (day)	
Cultivar	Egg	Larva	Pupa	Preadult	Female	Male
SM (Bell Pepper)	2.19 ± 0.0 a	3.78 ± 0.1 ab	7.53 ± 0.0 ab	13.5 ± 0.1 b	6.31 ± 0.2 a	4.27 ± 0.3 a
California Wonder (Bell Pepper)	2.16 ± 0.0 a	3.69 ± 0.1 ab	7.49 ± 0.0 ab	13.34 ± 0.2 b	6.45 ± 0.3 a	4.66 ± 0.3 a
Longo (Eggplant)	2.07 ± 0.0 b	3.60 ± 0.1 b	7.16 ± 0.0 b	12.83 ± 0.3 b	6.81 ± 0.2 a	4.78 ± 0.2 a
Kemer (Eggplant)	2.24 ± 0.0 a	4.02 ± 0.0 a	7.85 ± 0.0 a	14.11 ± 0.1 a	6.16 ± 0.2 b	4.09 ± 0.2 b

Means within each column followed by different letters are significantly different (paired bootstrap test; *p* < 0.05).

**Table 4:** Mean (±SE) reproductive attributes of *Aphidoletes aphidimyza* fed on *Myzus persicae* reared on different cultivars of bell pepper and eggplant plants under laboratory conditions.

	Reproduction			
Cultivar	TPOP(day)	APOP (day)	Oviposition Period (day)	Fecundity (Egg/Female)
SM (Bell Pepper)	14.68 ± 0.2 ab	1.36 ± 0.1 ab	4.63 ± 0.2 ab	21.05 ± 2.0 b
California Wonder (Bell Pepper)	14.26 ± 0.2 b	1.36 ± 0.1 ab	5.15 ± 0. 2 a	24.30 ± 2.2 ab
Longo (Eggplant)	13.59 ± 0.1 c	1.18 ± 0.1 b	5.31 ± 0.2 a	27.24 ± 2.a
Kemer (Eggplant)	15.22 ± 0.2 a	1.55 ± 0.1 a	4.21 ± 0.2 b	19.55 ± 1.9 b

TPOP, total pre-oviposition period; APOP, adult pre-oviposition period. Means within a column followed by different letters are significantly different (paired bootstrap test: *p* > 0.05).

**Table 5:** Mean (±SE) life table parameters of *Aphidoletes aphidimyza* fed on *Myzus persicae* reared on four different cultivars of bell pepper and eggplant under laboratory conditions.

Parameter (Mean ± SE)				
Cultivar	*R*_0_ (Offspring)	*r* (day ^−1^)	*λ* (day ^−1^)	*T* (day)
SM (Bell Pepper)	8.00 ± 1.6 a	0.120 ± 0.001 ab	1.127 ± 0.001 ab	17.13 ± 0.2 a
California Wonder	9.73 ± 1.8 a	0.134 ± 0.001 ab	1.144 ± 0.001 ab	16.72 ± 0.2 ab
(Bell Pepper)				
Longo (Eggplant)	11.98 ± 2.1 a	0.150 ± 0.001 a	1.162 ± 0.001 a	16.34 ± 0.1 b
Kemer (Eggplant)	7.03 ± 1.5 a	0.110 ± 0.001 b	1.117 ± 0.001 b	17.37 ± 0.2 a

Means within each column followed by different letters are significantly different (paired bootstrap test; *p* < 0.05). Development, survival, and fecundity of *C. carnea*. *R*_0_: net reproductive rates; *r*: intrinsic rate of increase; *λ*: finite rate of increase; *T*: generation time.

**Table 6:** Mean (±SE) developmental times (days) of immature and adult stages of *Chrysoperla carnea* fed on *Myzus persicae* reared on four different cultivars of bell pepper and eggplant.

	Immature Developmental Time (day)				Adult Longevity (day)	
Cultivar	Egg	Larva	Pupa	Preadult	Female	Male
SM (Bell Pepper)	3.42 ± 0.1 ab	11.27 ± 0.2 a	8.57 ± 0.0 ab	23.27 ± 0.2 a	59.42 ± 2.3 b	52.53 ± 2.7 b
California Wonder (Bell Pepper)	3.23 ±0.1 bc	8.83 ± 0.1 c	7.99 ± 0.0 c	20.05 ± 0.2 c	59.65 ± 3.6 ab	59.77 ± 3.4 a
Longo (Eggplant)	3.05 ± 0.1 c	10.64 ± 0.2 a	8.27 ± 0.1 bc	21.96 ± 0.3 b	65.99 ± 1.6 a	59.71 ± 1.9 a
Kemer (Eggplant)	3.61 ±0.1 a	9.77 ± 0.2 b	8.84 ± 0.1 a	22.22 ± 0.2 b	53.99 ± 2.6 b	56.21 ± 1.8 ab

Means within each column followed by different letters are significantly different (paired bootstrap test; *p* < 0.05).

**Table 7:** Mean (±SE) reproductive attributes of *Chrysoperla carnea* reared on *Myzus persicae* fed on four different cultivars of bell pepper and eggplant plants.

	Reproduction			
Cultivar	TPOP (day)	APOP (day)	Oviposition Period (day)	Fecundity (Egg/Female)
SM (Bell Pepper)	26.54 ± 0.2 a	3.55 ± 0.1 b	30.65 ± 1.5 b	577.70 ± 51.5 b
California Wonder (Bell Pepper)	23.35 ± 0.2 c	3.64 ± 0.2 ab	33.59 ± 3. 7 b	843.69 ± 59.6 a
Longo (Eggplant)	24.66 ± 0.3 b	3.56 ± 0.1 b	38.10 ± 1.6 a	958.12 ± 58.8 a
Kemer (Eggplant)	26.54 ± 0.2 a	3.55 ± 0.1 b	30.65 ± 1.5 b	577.70 ± 51.5 b

TPOP, total pre-oviposition period; APOP, adult pre-oviposition period. Means within a column followed by different letters are significantly different (paired bootstrap test: *p* > 0.05).

**Table 8:** Mean (±SE) life table parameters of *Chrysoperla carnea* reared on *Myzus persicae* fed on four different cultivars of bell pepper and eggplant under laboratory conditions.

Parameter (Mean ± SE)				
Cultivar	*R*_0_ (Offspring)	*r* (day ^−1^)	*λ* (day ^−1^)	*T* (day)
SM (Bell Pepper)	242.62 ± 45.53 ab	0.144 ± 0.005 ab	1.155 ± 0.006 ab	37.84 ± 0.5 a
California Wonder	286.89 ± 68.29 ab	0.159 ± 0.007 a	1.173 ± 0.008 a	35.21 ± 0.7 b
(Bell Pepper)				
Longo (Eggplant)	344.49 ± 68.12 a	0.157 ± 0.006 a	1.170 ± 0.007 a	36.93 ± 0.5 ab
Kemer (Eggplant)	175.12 ± 37.74 b	0.139 ± 0.006 b	1.149 ± 0.007 b	36.86 ± 0.5 ab

Means within each column followed by different letters are significantly different (paired bootstrap test; *p* < 0.05). *R*_0_: net reproductive rates; *r*: intrinsic rate of increase; *λ*: finite rate of increase; *T*: generation time.

**Table 9:** Mean (±SE) secondary metabolites contents aphid-infested and non-infested leaf extracts of each cultivar of bell pepper and eggplant (for each cultivar individually).

	Phenol (mgGAE/gfw)		Flavonoid (mgQE/gfw)		Anthocyanin (mgCE/gDP)	
Cultivar	Non-Infested	Infested	Non-Infested	Infested	Non-Infested	Infested
SM (Bell Pepper)	68.8 ± 2.4 B,b	104.2 ± 6.8 AB,a **	92.2 ± 5.4 B,a	118.8 ± 11.7 C,a	0.6 ± 0.0 B,b	1.1 ± 0.0 A,a **
California Wonder (Bell Pepper)	95.3 ±6.8 A,a	136.0 ± 18.5 A,a	148.7 ± 5.7 A,b	225.3 ± 19.6 A,a **	0.5 ± 0.0 B,b	1.0 ± 0.0 A,a **
Longo (Eggplant)	109.3 ± 2.1 A,b	125.4 ± 1.1 AB,a **	170.4 ± 9.3 A,b	201.9 ± 2.6 AB,a *	0.9 ± 0.0 A,b	1.2 ± 0.0 A,a **
Kemer (Eggplant)	55.2 ± 4.0 B,b	86.2 ± 3.0 B,a **	68.9 ± 1.6 B,b	118.4 ± 3.0 C,a **	0.4 ± 0.0 B,b	0.7 ± 0.0 B,a *

Means within each row followed by different lowercase letters are significantly different (* *p* < 0.05, ** *p* < 0.01, *t* test). Means within each column followed by different uppercase letters are significantly different (Tukey’s test, *p* < 0.05).

**Table 10:** Mean (±SE) enzyme activities in aphid-infested and non-infested leaf extracts of each cultivar of bell pepper and eggplant.

	PPO (µmol/mg protein/min)		SOD (µmol/mg protein/min)		CAT (µmol/g fw/min)	
Cultivar	Non-Infested	Infested	Non-Infested	Infested	Non-Infested	Infested
SM (Bell Pepper)	0.003 ± 0.0 AB,b	0.006 ± 0.0 C,a *	2501 ± 92.1 BC,b	3615 ± 22.7 C,a **	0.071 ± 0.0 AB,b	0.125 ± 0.0 B,a *
California Wonder (Bell Pepper)	0.002 ± 0.0 B,b	0.007 ± 0.0 BC,a **	2587 ± 84.0 B,b	4056 ± 27.5 B,a **	0.091 ± 0.00 AB,b	0.149 ± 0.0 B,a **
Longo (Eggplant)	0.006 ± 0.0 A,b	0.015 ± 0.0 A,a *	3552 ± 86.7 A,b	5003 ± 62.1 A,a **	0.125 ± 0.00 A,b	0.198 ± 0.0 A,a **
Kemer (Eggplant)	0.005 ± 0.0 ABb	0.013 ± 0.0 AB,a *	2210 ± 62.1 C,b	3337 ± 32.7 D,a **	0.057 ± 0.00 B,b	0.086 ±0.0 C,a *

Means within each row followed by different lowercase letters are significantly different (* *p* < 0.05, ** *p* < 0.01, *t* test). Means within each column followed by different uppercase letters are significantly different (Tukey’s test, *p* < 0.05).

## Data Availability

The original contributions presented in this study are included in the article. Further inquiries can be directed to the corresponding author.
